# ﻿A new nothogenus and nothospecies in the Zygopetalinae (Orchidaceae), with a note on euglossine pollination in the subtribe

**DOI:** 10.3897/phytokeys.266.159892

**Published:** 2025-11-07

**Authors:** Franco Pupulin, Adam P. Karremans, Elvira Salas Hidalgo

**Affiliations:** 1 Lankester Botanical Garden, University of Costa Rica, P.O.Box 302-7050 Cartago, Costa Rica University of Costa Rica Cartago Costa Rica; 2 Harvard University Herbaria, Cambridge, MA, USA Harvard University Herbaria Cambridge United States of America; 3 The Marie Selby Botanical Gardens, Sarasota, FL, USA The Marie Selby Botanical Gardens Sarasota United States of America; 4 Department of Biochemistry, School of Medicine, University of Costa Rica, San José, Costa Rica University of Costa Rica San José Costa Rica

**Keywords:** *Benzingia* pollinaria, ×*Benzoscaphe*, *Chondroscaphe* pollinaria, natural hybridization, orchid hybrid pollinarium

## Abstract

Natural hybridization in Neotropical orchids is summarized, and the frequency of nothogenera in the subtribe Zygopetalinae is highlighted. A new nothogenus discovered in Costa Rica, formed by the cross between species of *Benzingia* and *Chondroscaphe*, is described, and the new nothospecies ×*Benzoscaphe stelleri* is described and illustrated. Putative parents, as well as their derivative hybrid, are compared through composite digital plates. Cues are offered about the pollination mechanisms of the putative parents, based on the morphology of the flowers and pollinaria, which possibly led to the formation of the natural cross between different genera.

## ﻿Introduction

Hybrid taxa formed by a natural cross between species of the same genus have been recorded in several orchid groups in the Neotropics. Individuals belonging to a nothotaxon are not easy to detect, and their presence is usually documented on the basis of unique combinations in the morphology of plants and flowers, which present intermediate traits between already known sympatric species, whether in the same or in different genera. Nothospecies are known in subfamily Cypripedioideae (*Cypripedium*: Szlachetko et al. 2017; *Phragmipedium*: Pupulin and Díaz-Morales 2018; Díaz-Morales and Pupulin 2019), subfamily Vanilloideae (*Vanilla*: Pansarin 2025), and in many subtribes in the Epidendroideae. Among them, natural hybrids have been documented in Catasetinae (*Catasetum*: Cantuária et al. 2021; Romero-González and Carnevali 2024; Krahl et al. 2024), Dendrobiinae (*Bulbophyllum*: Borba and Semir 1998; Azevedo et al. 2006; Fiorini et al. 2023), Cyrtopodiinae (*Cyrtopodium*: Batista and Medeiros 2022), Laeliinae (*Cattleya*: Braem 1995; Castro Neto and Catharino 2004; van den Berg 2014; Barrera-Rojas and van den Berg 2025; *Encyclia*: Dressler and Pollard 1974; Sauleda and Adams 1990; Pérez-García and Hágsater 2012; Sauleda and Esperón 2016, 2019; León-Peralta et al. 2023; *Epidendrum*: Pupulin 2003; Pinheiro et al. 2010; Bogarín et al. 2011; *Laelia*: Salazar et al. 2014; Solano et al. 2019; Cetzal-Ix et al. 2020; *Prosthechea*: Mó et al. 2014; *Tetramicra*: Soto Calvo et al. 2019), Oncidiinae (*Cohniella*: Cetzal-Ix et al. 2013; *Ionopsis*: Krahl et al. 2021; *Lophiaris*: Cetzal-Ix et al. 2018), Pleurothallidinae (*Dracula*: Zambrano and Solano-Gómez 2011; *Masdevallia*: Lipińska et al. 2019; Archila Morales et al. 2021; *Pleurothallis*: Pupulin et al. 2021), and Stanhopeinae (*Gongora*: Jiménez-Vázquez et al. 2023).

Less frequent are hybrid taxa formed between species belonging to different genera. Among the Oncidiinae, natural hybrids between *Comparettia* and *Rodriguezia* were reported from Colombia (Sauleda 2015; Sauleda and Arrondeau 2020), while Cetzal-Ix et al. (2012) described the new nothogenus ×*Cohnlophiaris*, resulting from the cross between species of *Cohniella* and *Lophiaris*. However, according to alternative generic circumscriptions (i.e., Neubig et al. 2012), the latter nothogenus is considered a synonym of *Trichocentrum*. Chiron and Castro Neto (2002) described the hybrid genus ×*Hoffmanncattleya*, which includes natural hybrids between the genera *Cattleya* and *Hoffmannseggella* (Castro Neto and Menezes 2007). However, most authors consider the two genera synonymous.

Compared to the paucity of recorded intergeneric hybrids among other orchid groups, the Zygopetalinae appears particularly interesting, with at least four nothospecies recorded in three natural nothogenera: ×*Bensteinia* Christenson (Christenson 2006; Pupulin 2007), ×*Cochlezella* J.M.H.Shaw (Pupulin 2015), and ×*Pescatoscaphe* Christenson (Christenson 2006). Here, we record another nothogenus in the subtribe, ×*Benzoscaphe*, resulting from the cross between *Benzingia reichenbachiana* (Schltr.) Dressler and a species of *Chondroscaphe* (Dressler) Senghas & G. Gerlach, which we hypothesize to be *C. bicolor* (Rolfe) Dressler. Interestingly, the hybrid between *Benzingia* Dodson and *Chondroscaphe* has never been produced artificially. The first genus has been hybridized with *Kefersteinia* Rchb.f. to produce ×*Bensteinia*, with two natural nothospecies occurring in Ecuador and Costa Rica (Christenson 2006; Pupulin 2007), and with *Cochleanthes* Raf. plus *Kefersteinia* to create the hybrid swarm *Goldnerara* (Royal Horticultural Society 2017). *Chondroscaphe* has been artificially crossed with *Pescatoria* Rchb.f. (= ×*Pescatoscaphe*, which today also includes the hybrids of the synonymous *Bollea* Lindl., previously treated as ×*Bolleoscaphe*), *Cochleanthes* (= ×*Cochloscaphe*), *Warscewiczella* Rchb.f. (= ×*Warczewscaphe*), and *Zygopetalum* Hook. (= ×*Zygoscaphe*) (Royal Horticultural Society 2017).

When the astonishing size of the Orchidaceae is taken into account, likely exceeding 30,000 species (Kindlmann et al. 2023), the number of recorded naturally occurring hybrids is remarkably low. Nevertheless, multiple factors contribute to the paucity of nothotaxa reported to date. Orchids possess a complex system of barriers, both abiotic and biotic, to prevent pollen from being deposited on the female reproductive organs of the “wrong” species. Among the abiotic barriers, the most significant are undoubtedly the geographic isolation between species whose reproductive systems are mechanically compatible and the temporal barrier resulting from a chronological shift in the phenology of morphologically related species. The latter scenario is especially true in groups whose flowers are “generalist” in attracting pollinators, as is the case, for example, in the genus *Sobralia* and other groups characterized by ephemeral flowers and synchronous flowering. If these barriers are disrupted—as sometimes happens due to anthropogenic transformation of the natural landscape—other factors related to the biology, morphology, mechanics, and chemistry of flowers come into play to reduce the possibility of erroneous gamete exchange and to ensure that the genetic coherence of the species is maintained. Species-specific attractive signals, precise shape and size of floral organs to limit interaction exclusively to the correct pollinator, highly specific positioning of the pollinarium, and its morphological and structural transformation after removal aimed at precisely orienting the pollen toward the stigmatic cavity are some of the functional strategies that orchids employ to prevent erroneous sexual crossing (Tremblay et al. 2005; Ackerman et al. 2023). When these are also thwarted by an error in the pollen vector, other chemical barriers are put in place to maintain species identity, such as incompatible signals that compromise the activation of pollen tubes or, ultimately, genetic incompatibility between sperm cells and the ovules that should be fertilized.

Considering the large number and complex nature of the barriers that prevent interbreeding between different species, it is notable that they can be evaded in groups belonging to all the subfamilies of Orchidaceae recorded in the Neotropics. Documenting a naturally occurring interspecific or, even more so, intergeneric hybrid represents an event with infinitesimal statistical probability. In the field, this means encountering a hybrid individual that vegetatively closely resembles one or both of its parents, likely a unique individual that—due to its unique floral morphology—may not have left any offspring, collecting that individual entirely by chance, cultivating it to flower, and having prior knowledge of the putative species that may have generated it, allowing comparison and thus advancing a scientific hypothesis regarding its hybrid nature. Seen in this way, it is perhaps not too speculative to suppose that the relatively few nothospecies documented so far represent nothing more than the tip of an iceberg of hybrid individuals that coexist in nature with their ancestors. From an evolutionary perspective, the distinction between F1, subsequent filial generations, as well as backcrosses and combinations of these could be of potential interest, even though for the International Code of Nomenclature for algae, fungi, and plants (Turland et al. 2025), a nothotaxon includes all individuals derived from the crossing of representatives of the stated parent taxa (Art. H.11.1).

Studying the role of natural hybridization in orchid evolution has certainly been hampered to date by the limited number of individuals with diagnosable morphological characteristics that allow their identification as such. More generally, the sporadic introduction of new genetic combinations into a population could lead to increased genetic variability, while repeated backcrossing could result in polyploidy and favor the incorporation of genes that accelerate adaptation.

During the systematic documentation of Costa Rican species of Orchidaceae, mostly intended for the treatment of the family for *Flora Costaricensis* (Atwood and Mora-Retana 1999; Pupulin 2010), we have received considerable help from a large group of resident naturalists and plant enthusiasts, who share with the personnel of Lankester Botanical Garden their most unusual findings. This kind of cooperation, which in the recent history of our research center has made possible the discovery and scientific description of several rare and rarely seen orchid species for the flora of Costa Rica, is a true example of what citizen science can accomplish when a local scientific institution opens its doors to the contributions of trained naturalists. In the specific case of the new nothospecies described in this paper, one of the plants from a collection made on the southeastern slopes of the Central Volcanic Cordillera of Costa Rica was grown and flowered by Norman Steller, a keen orchid grower who collected the plant together with staff of Lankester Botanical Garden and brought it to our research department for documentation and study.

## ﻿Material and methods

The plants used for this study were collected during routine field activities carried out by the scientific and horticultural staff of Lankester Botanical Garden (JBL, by its acronym in Spanish) under the scientific permit R-SINAC-SE-DT_PI-005-2024 issued by the Costa Rican Ministry of Environment and its National System of Protected Areas (SINAC, by its acronym in Spanish). The plants were cultivated in the research greenhouses of JBL and in private greenhouses until flowering, for documentation and study. Macro photographs were taken with Nikon D850 and D810 cameras fitted with a Micro-Nikkor AF 105 mm lens. Photomicrographs were taken with a Leica Z16 zoom microscope fitted with a Planapo 1× lens and recorded on a Nikon D810 camera via a Leica trinocular tube and dedicated photo tubes for a full-frame format sensor. Black-and-white line drawings were made on Apple iPad 9^th^ generation tablets using the Procreate 5.3.15 raster graphics editor, laser-printed, and rendered with Rapidograph technical pens. As this nothospecies—as with most of the Zygopetalinae belonging to the “*Chondrorhyncha* complex”—produces only a single, subapical flower per inflorescence, the dissected parts of the perianth were stored in FAA to serve as type material. The voucher was then accessioned into the liquid collection of the Lankester Botanical Garden Herbarium (JBL). The original plant from which the type material was prepared is maintained in cultivation, in the hope that it can produce more flowers in the future. Even though these flowers will not have any significance in terms of typification (being prepared on a date different from that of the holotype, they do not belong to the original materials), they would nonetheless be a useful reference, as they originate from the same individual plant that served as the type. The plant and flower of the new nothospecies were compared with living material grown in the Center’s greenhouses and with conserved flowers in the JBL herbarium of both putative parental species. Data on the distribution range of the putative parents were extracted from the extensive georeferenced databases of orchid distribution maintained at the Center, and the resulting habitat-compatible regions were plotted on an orographic map of Costa Rica (see further under the section on Distribution). Phenological data from both field-collected and cultivated plants of the putative parents were gathered and examined to test the consistency of the hypothesis. FP conceptualized the paper; FP and APK wrote the original draft; and ES wrote the descriptions and reviewed and edited the manuscript.

## ﻿Taxonomy

### ﻿× Benzoscaphe

Taxon classificationPlantaeAsparagalesOrchidaceae

Pupulin nothogen. nov.

F63D5003-A7C9-54F2-AEF3-A9082F7BD110

urn:lsid:ipni.org:names:77371638-1

#### Type species.

×*Benzoscaphe stelleri* Pupulin, Karremans & E.Salas

#### Diagnosis.

Nothogenus novum caracteribus intermediis inter Benzingiam *Dodson* et Chondroscaphen *(Dressler) Senghas et G.Gerlach*. Folia viridi-grisacea sicut Benzingiarum species sed erecta prod pendulis sicut Chondroscapharum species; sepalis lateralibus omnino reflexis subuncinatis, basi petalorum ad columna adnata, pede columnae elongato Benzingiae similiter; inflorescentia suberecta prod pendula, petalis subtrapezoideis, labello callis duobus ornato, anthera elongata anguste ovata, polliniis elongatis valde curvatis supra stipitem rectangulo concavo-convexo viscidioque incrassato sicut Chondroscapharum species; floris magnitudo et forma labelli intermediis inter genera dignoscenda.

#### Description.

Epiphytic, caespitose, erect ***herbs*** with a tuft of leaves fan-like arranged. ***Roots*** slender, flexuous, whitish. ***Leaves*** narrowly elliptic, acuminate, with a short conduplicate petiole, grayish green, articulated with basal, ovate, conduplicate sheaths provided with hyaline margins. ***Inflorescence*** a single-flowered raceme, suberect, much shorter than the leaves. ***Floral bracts*** two, the outer bract larger, broadly ovate, loose, the inner bractlet lanceolate, smaller. Pedicellate ovary terete, gently curved toward the apex. ***Flower*** spreading, downward facing, the ***dorsal sepal*** porrect-recurved, the ***lateral sepals*** reflexed, subuncinate, the ***petals*** inserted along the basal side of the column, porrect with the apex gently recurved, the ***lip*** obscurely three-lobed, the lateral lobes erect, the midlobe geniculate-bent, with ruffled margins; disc with a median, irregularly dentate callus and a second thickening toward the apex of the midlobe. ***Column*** hemiterete, dilated around the stigma, provided with a long foot, the stigma transversal, slit-like, the clinandrium swallow, the ***anther*** ovate; ***pollinia*** 4 in two superposed pairs of different sizes, on a rectangular, erected stipe and a narrowly lanceolate, thick viscidium.

**Figure 1.**
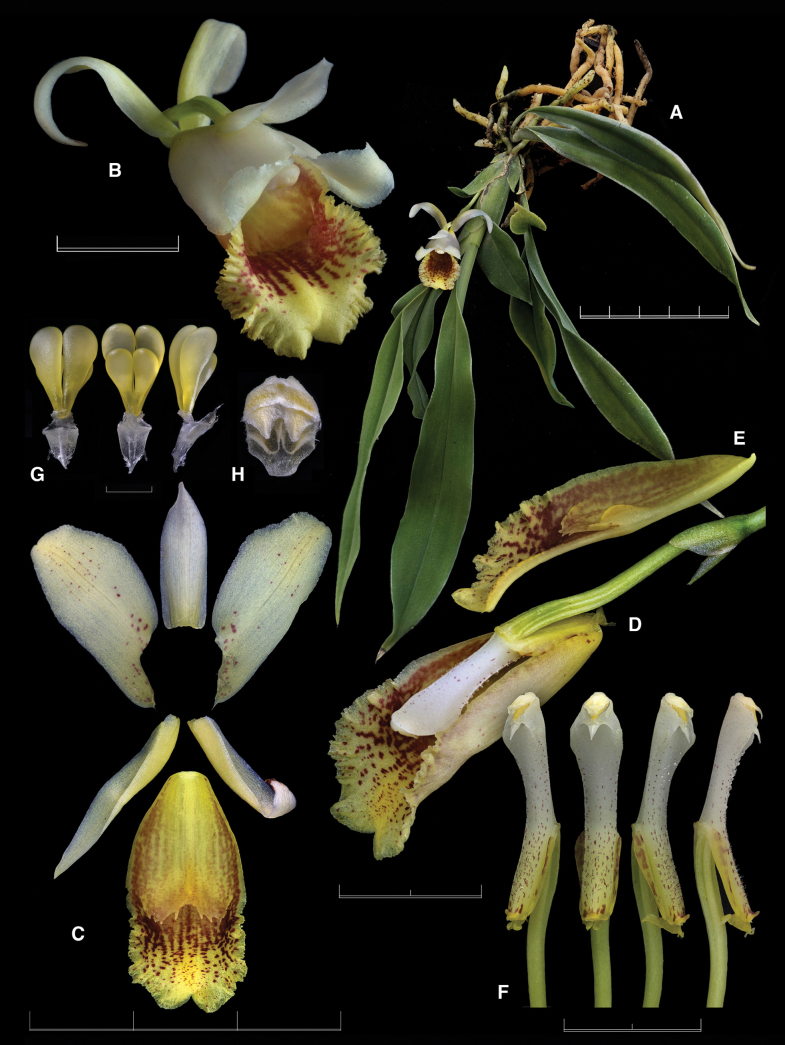
Lankester Composite Digital Plate of *Benzingia reichenbachiana*. **A.** Habit; **B.** Flower; **C.** Dissected perianth; **D.** Ovary, column, and lip in three-quarters view; **E.** Lip in longitudinal section; **F.** column, several views; **G.** Pollinarium in dorsal, ventral, and lateral views: **H.** anther cap. Scale bars: 1 mm (**G, H**); 2 cm (**D, E, F**); 3 cm (**C**); 5 cm (**A**). Prepared by F. Pupulin from *Pupulin 5159* (voucher at JBL-B0354).

**Figure 2.**
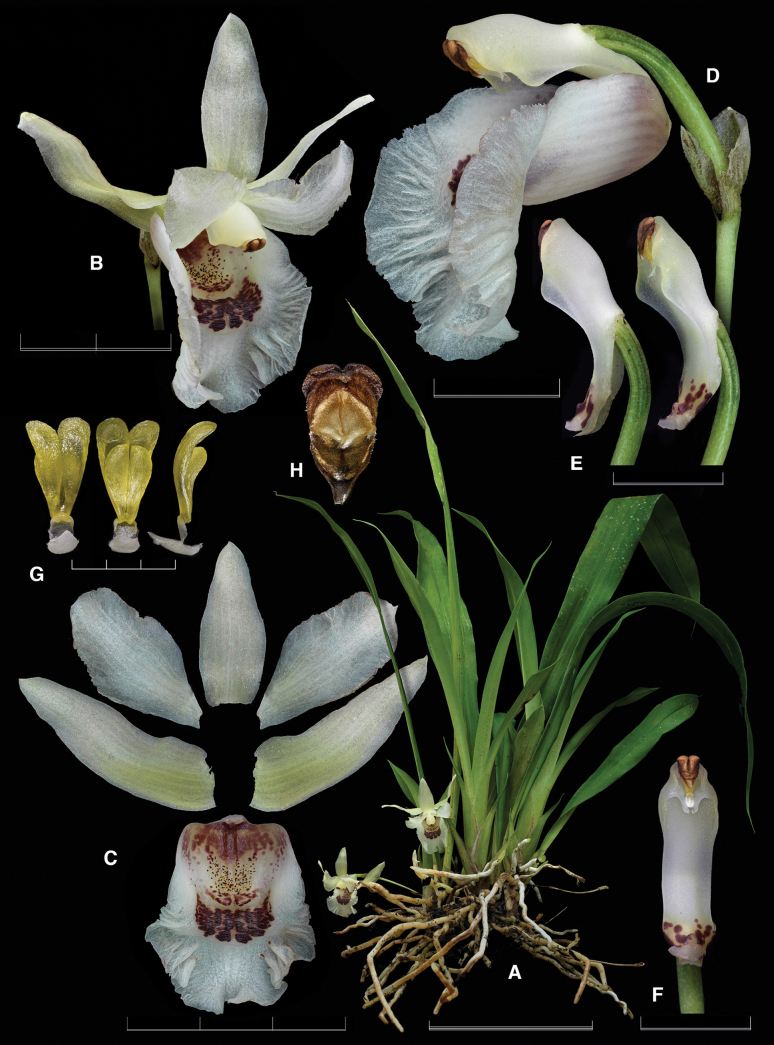
Lankester Composite Digital Plate of *Chondroscaphe bicolor*. **A.** Habit; **B.** Flower; **C.** Dissected perianth; **D.** Ovary, column, and lip in three-quarters view; **E.** Column in lateral and three-quarters views; **F.** Column in ventral view; **G.** Pollinarium in dorsal, ventral, and lateral views; **H.** Anther cap. Scale bars: 3 mm (**G, H**); 1 cm (**D, E, F**); 2 cm (**B**); 3 cm (**C**); 10 cm (**A**). Prepared by F. Pupulin from *Bogarin 14717* (voucher at JBL-L0130).

The new nothogenus presents intermediate features between the genera *Benzingia* (Fig. 1) and *Chondroscaphe* (Fig. 2). The leaves are grayish green with the epidermis somewhat prismatic, like in *Benzingia*, but they are held erect as in species of *Chondroscaphe*. The size of the flower is intermediate between the smaller flower of *Benzingia* (natural spread of flower *ca.* 3 cm) and larger ones typical of *Chondroscaphe* (spread flowers >6 cm). The flower of the nothogenus is similar to that of *Benzingia* in the lateral sepals completely reflexed and uncinate at the apex, the base of the petals adnate to the margins of the column, and the elongate column foot. It resembles *Chondroscaphe* in the subtrapezoid petals, the lip provided with a basal callus and a distinct apical bulging, the narrowly ovate, elongate anther cap, and the shape of the pollinarium, with four elongate, distinctly curved pollinia lying over a rectangular, concave-convex stipe, attached to a thick viscidium.

### ﻿× Benzoscaphe stelleri

Taxon classificationPlantaeAsparagalesOrchidaceae

Pupulin, Karremans & E.Salas nothosp. nov.

502E5546-3A73-5869-920D-EAA98F583823

urn:lsid:ipni.org:names:77371639-1

#### Type.

**Figure 3.**
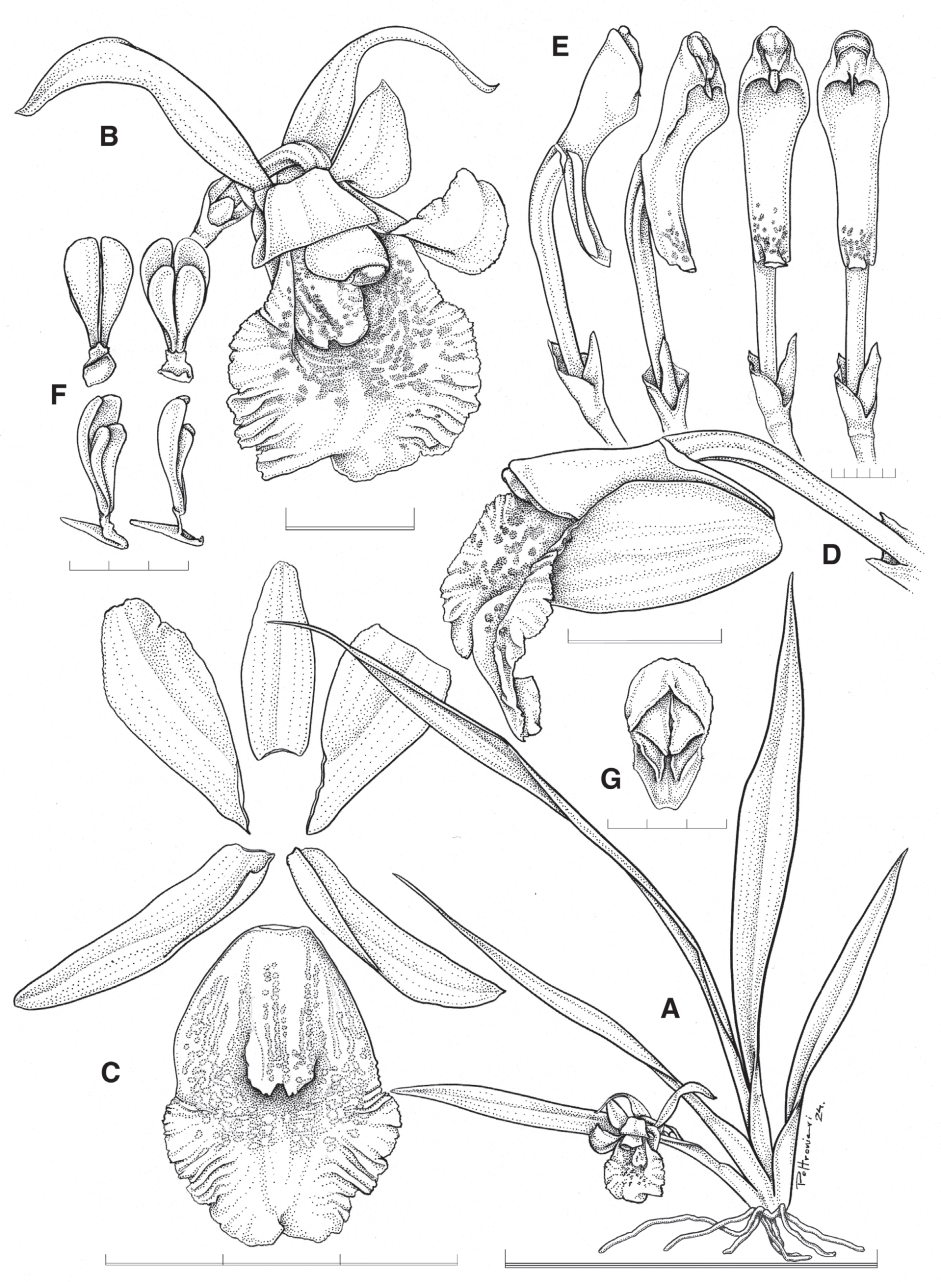
×*Benzoscaphe stelleri.***A.** Habit **B.** Flower **C.** Dissected perianth; **D.** Ovary, column, and lip in three-quarters view; **E.** Column in lateral, three-quarters, and ventral views (emasculated at the right); **F.** Pollinarium in dorsal, ventral, three-quarters, and lateral views; **G.** Anther cap. Scale bars: 3 mm (**F, G**); 5 mm (**E**); 1 cm (**B, D**); 3 cm (**C**); 10 cm (**A**). Drawn by S. Poltronieri from *F. Pupulin 9146* (voucher at JBL-K0409).

**Figure 4.**
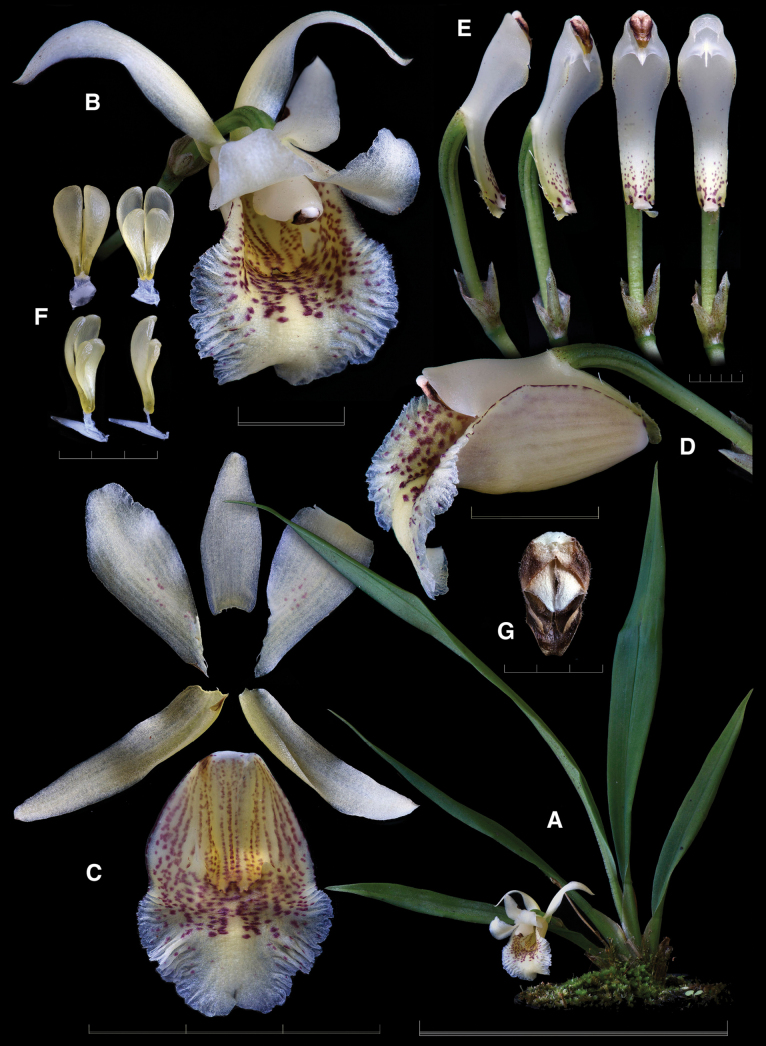
Lankester Composite Digital Plate of ×*Benzoscaphe stelleri.***A.** Habit; **B.** Flower; **C.** Dissected perianth; **D.** Ovary, column, and lip in three-quarters view; **E.** Column in lateral, three-quarters, and ventral views (emasculated at the right); **F.** Pollinarium in dorsal, ventral, three-quarters, and lateral views; **G.** Anther cap. Scale bars: 3 mm (**F, G**); 5 mm (**E**); 1 cm (**B, D**); 3 cm (**C**); 10 cm (**A**). Prepared by F. Pupulin from *F. Pupulin 9146* (voucher at JBL-K0409).

**Figure 5.**
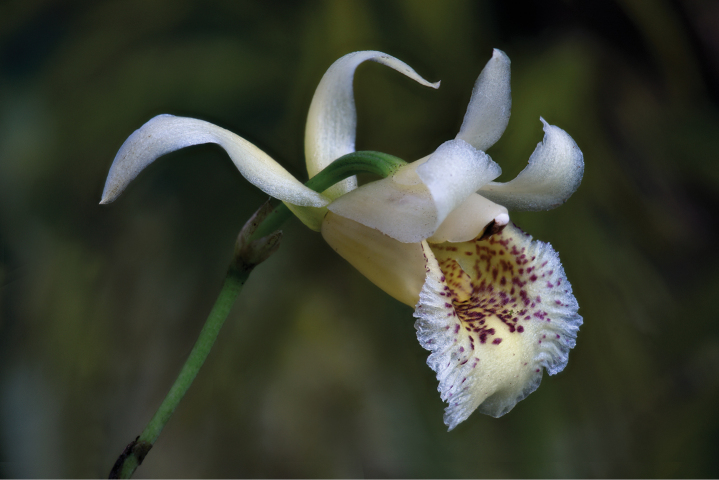
Flower of ×*Benzoscaphe stelleri*, from the plant that served as the holotype. Photograph by F. Pupulin.

Costa Rica • Alajuela: Naranjo, San Juanillo, road to Zarcero, epiphytic in forest remnants at the right of the road, 10°08'40.4"N, 84°23'13.0"W, 1450 m, 14 March 2024, *F. Pupulin 9146 & N. Steller* (***holotype***, JBL-K0409). (Figs 3–5).

#### Diagnosis.

Nothospecies nova caracteribus intermediis inter Benzingiam reichenbachianam (*Schltr.*) *Dressler* et valde probabiliter Chondroscaphen bicolorem *(Rolfe) Dressler* dignoscenda. Folia viridi-grisacea sicut Benzingiae reichenbachianae sed erecta prod pendulis sicut Chondroscaphe bicolor; sepalis lateralibus omnino reflexis subuncinatis, basi petalorum ad columna adnata, pede columnae elongato Benzingiae similiter; inflorescentia suberecta prod pendula, petalis subtrapezoideis, labello callis duobus ornato, anthera elongata anguste ovata, polliniis elongatis valde curvatis supra stipitem rectangulo concavo-convexo viscidioque incrassato sicut Chondroscaphe bicolor; floris magnitudo et forma labelli intermediis inter duabus speciebus.

#### Description.

Epiphytic, caespitose, erect ***herbs*** with a tuft of 4–5 leaves arranged like a fan. ***Roots*** flexuous, slender, ca. 1 mm in diameter, whitish, the apex green. ***Leaves*** narrowly elliptic, acuminate, 8.0–20.0 × 1.0–1.8 cm, with a short conduplicate petiole to 0.5 cm long, grayish green, paler on the underside, articulated with basal, ovate, strongly conduplicate sheaths 1.4–3.0 cm long, the margins scabrous-hyaline. ***Inflorescence*** a single-flowered raceme, suberect, to 5 cm long; peduncle terete, slender, ca. 3 cm long. ***Floral bracts*** two, the outer bract larger, broadly ovate, loose, 7 × 6 mm, the inner bractlet lanceolate, smaller, 7 × 2 mm. Pedicellate ***ovary*** terete-subclavate, gently curved toward the apex, 2 cm long. ***Flower*** spreading, downward facing, the sepals and petals creamy white, the petals marked with few, scattered rose-purple spots near the base, the lip white, flushed pale yellow toward the base, with lines of purple spots (nectar guides) running from the base to about two-thirds of the blade, the callus on the disc yellow, the column white, sparsely dotted with purple-red at the base, the anther cap cream to light brown. ***Dorsal sepal*** lanceolate, acute, 15 × 7 mm, porrect-recurved. ***Lateral sepals*** narrowly lanceolate, acute, abruptly apiculate, 25 × 5 mm, strongly reflexed, subuncinate at apex, the labellar margin enrolled from the base to the middle. ***Petals*** inserted along the basal side of the column, obliquely obovate to narrowly subrhombic, truncate, 22 × 8 mm, porrect, the apex gently recurved. ***Lip*** obscurely three-lobed, 28 × 21 mm, the lateral lobes elliptic, erect to flank the column, the midlobe broadly ovate-trapezoid, apically bilobed, geniculate-bent, apically shortly reflexed, with ruffled margins; disc with a median, irregularly dentate callus, slightly elevated callus in the middle of laminate, and a second thickening toward the apex of the midlobe. ***Column*** hemiterete, ca. 10 mm long, dilated around the stigma into elliptic-subtriangular wings, 5 mm wide at the broadest point, provided with a long foot ca 10 mm long, the stigma transversal, slit-like, the rostellum acicular, flanked by 2 pararostellar teeth, the clinandrium low-cuspidate, swallow. ***Anther cap*** ovate, apically refuse, 2-celled. ***Pollinia*** 4, narrowly ovate, complanate, in two superposed pairs of different sizes, on a short, rectangular, erected stipe and a narrowly lanceolate, thick viscidium.

#### Distribution.

**Figure 6.**
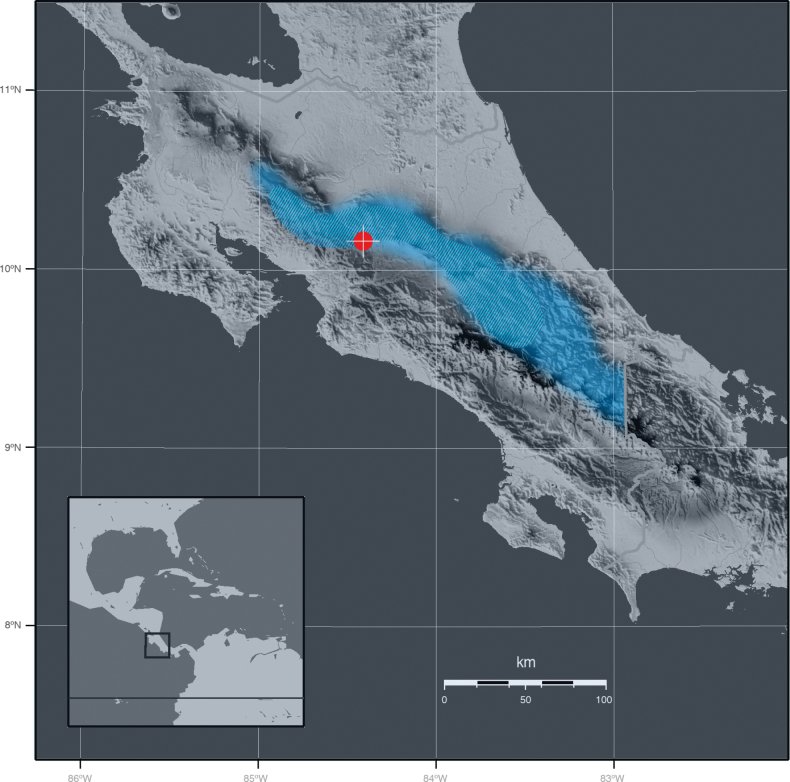
Map showing the type locality of ×*Benzoscaphe stelleri* (red circle) and the known distribution ranges in Costa Rica of *Chondroscaphe bicolor* (light blue) and *Benzingia reichenbachiana* (lined blue).

Only known from central Costa Rica, where it has been so far documented only from about 1500 meters of elevation along the southeastern slopes of the Central Volcanic Cordillera, facing the Central Valley (Fig. 6).

#### Habitat and phenology.

The nothospecies inhabits the premontane wet forests of the geologically recent Central Volcanic Cordillera in Costa Rica, where it has been exclusively found along the slopes of the range facing south, a region mostly influenced by the Pacific climate. Flowers are produced at least in February and March, at the beginning of the dry season in Costa Rica.

#### Eponymy.

Dedicated to Norman Steller, of Naranjo, Costa Rica, who participated in the collection of the plant that served as type.

The plant and flower of *Benzoscaphe stelleri* appear to have no morphologically similar taxa in Costa Rica. Both vegetatively and in terms of floral morphology, its characteristics appear to be intermediate between those of *Benzingia reichenbachiana* and (most likely) *Chondroscaphe bicolor*. The leaves of the nothospecies are grayish-green in color, with a prismatic-appearing epidermis, as in *B. reichenbachiana*, but they are erect and not wavy, as in the genus *Chondroscaphe*. As for the flower, the lateral sepals are completely reflexed and subuncinate at the apex (rather than spreading), the base of the petals is completely adnate to the column, and the latter has an elongate foot, as in *Benzingia* (short in *Chondroscaphe*). On the contrary, the suberect (rather than pendulous) inflorescence, the subtrapezoid petals, the labellum adorned with two rows of calli (only one central in *Benzingia*), the elongated and narrowly ovate anther, and the elongated and strongly curved pollinia, affixed to a concave-convex rectangular stipe and an enlarged viscidium, are similar to those of *Chondroscaphe*. The size of the flowers and the general shape of the labellum are intermediate between those of the two species.

We do not have conclusive evidence on the pollination of *Benzingia* Dodson species, but Gerlach and Schill (1991) suggest *B. palorae* (Dodson & Hirtz) Dressler (as *Stenia* Lindl.) is pollinated by male Euglossini bees, while Heide-Jørgensen (2021) reports that in the forests of the Ecuadorian Amazon *B. caudata* (Ackerman) Dressler (as *Chondrorhynhca* Lindl.) is pollinated by *Euglossa* sp., which receives the pollinarium on the head. Ramírez et al. (2002) reported, based on a personal observation by Robert Dressler, pollination of *B. reichenbachiana* (Schltr.) Dressler by *Euglossa heterosticta*, a relatively small bee ca. 1 cm long, but the authors gave no further details regarding the interaction between flower and insect and the place of pollinarium deposition. The same observation is reported by Lipińska and Barabasz (2024) in their review of pollination in Guatemalan orchids. The specific form of the pollinarium and its position on the insect’s body play a key role in effective fertilization of the flower and allow pollen deposition to be species-specific even among closely related taxa. Given the precise and highly specific size and shape of pollinarium and those of the foraging insects, as well as the ability of the flower to manipulate the insect’s behavior to place it in the correct position to receive and deposit the pollen, the margins for mechanical errors, possibly leading to pollination between different taxa, are limited. For example, within the subtribe Zygopetalinae, to which the species in this study belong, Nunes et al. (2017) demonstrated how in *Warrea* Lindl. and *Zygopetalum* the bee removes the pollinarium when getting out of the cavity formed by the lip and the column. In *Warrea*, however, the pollinarium is deposited on the thorax of *Bombus brasiliensis*, while in *Zygopetalum maxillare* G.Lodd. and *Z. crinitum* G.Lodd. it adheres to the back of the head and the head front, respectively, of the carpenter bees that visit the flowers.

**Figure 7.**
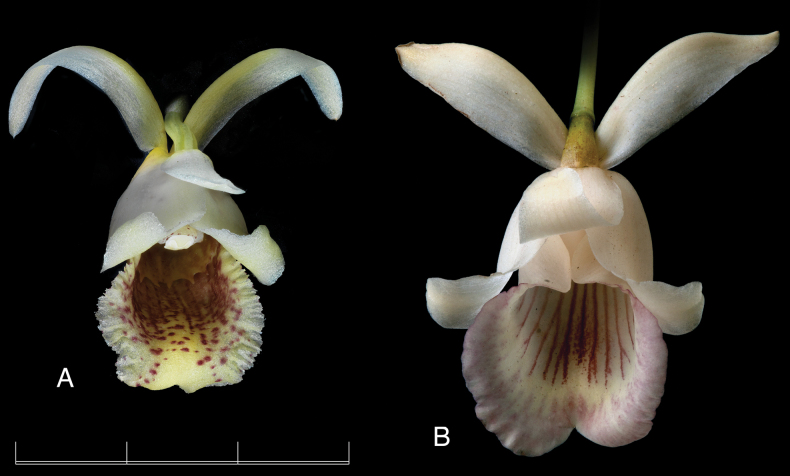
Flowers of Zygopetalinae with back-swept and inrolled lateral sepals. **A.***Benzingia reichenbachiana*; **B.***Warscewiczella lipscombiae*. Scale bar: 3 cm. Photographs by F. Pupulin.

There are elements in the morphology of the flower and pollinarium of *Benzingia* that are indicative of their possible general functioning. The flowers of *Benzingia reichenbachiana* are gullet-shaped, with the labellum spreading in front and the lateral sepals completely back-swept and inrolled, hooked towards the apex, so as to imitate nectaries (Fig. 7A). In a similarly shaped flower (*Warscewiczella lipscombiae*, Fig. 7B), Ackerman (1983) was able to observe several individuals of *Eulaema meriana*, *Euglossa cognata*, and *E. imperialis* extending their tongues into the cavity formed by the sepals in search of nectar. Most of these floral visitors are too small to come into contact with the column that overhangs the labellum, but Ackerman (1983) documented a female of *E. meriana* that, after extending the tongue into the nectar less nectary, received the pollinarium on the back of its head when backing out. The presence of “pseudo-nectaries,” as well as the broad and low laminar callus at the base of the labellum, appears in fact to be designed to allow the insect to advance deeply into the flower in search of a reward. In turn, the relatively large shield-like shape of the viscidium and the tegula that remains raised after removal suggests that the pollinarium is deposited on the head or on the frontal part of the scutellum of the visiting insect.

**Figure 8.**
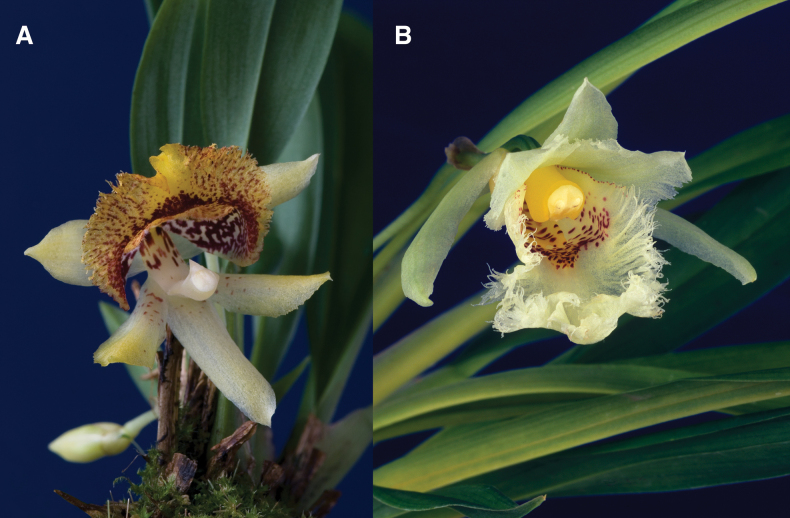
Anomalous flowers of Zygopetalinae. **A.***Benzingia hirtzii*, the flower non-resupinate; **B.***Chondroscaphe embreei*, the lateral sepals not back-swept. Photographs by F. Pupulin.

Nevertheless, it is possible that several similar, albeit different, pollination syndromes exist in *Benzingia*, as the flowers of *B. hirtzii* Dodson are non-resupinate (Fig. 8A), so that the visiting bee probably receives the pollinarium on the ventrum rather than on the front or the back of the body (Pupulin 2009). A similar placement on the ventral part of the insect’s body has been hypothesized for two species of *Kefersteinia* with non-resupinate flowers (Carnevali et al. 2015; Sierra-Ariza and Harding 2023), while in species with resupinate flowers the pollinarium is deposited at the base of one of the bee’s antennae (Dressler 1972; Pupulin 2009).

**Figure 9.**
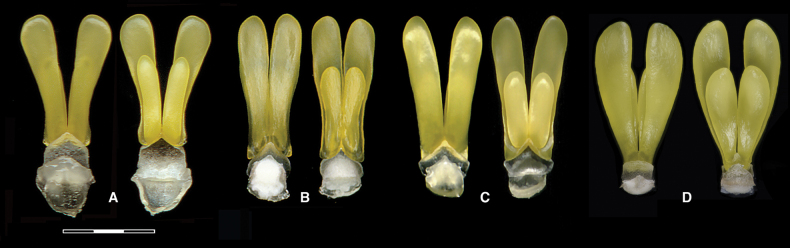
Pollinaria of *Chondroscaphe* species. **A.***C. embreei*; **B.***C. atrinlinguis*; **C.***C. yamilethiae*; **D.***C. bicolor*. Scale bar: 3 mm (**A–D**). Photographs by F. Pupulin.

Unfortunately, even though the pollinaria of *Chondroscaphe* (Dressler) Senghas & G. Gerlach (as *Chondrorhyncha* Lindl.) have been found on a male bee, *Euglossa asarophora*, records of pollination in species belonging to the genus are limited to observations of a single species (Reynolds 2004). Ackerman (1983) suggested that the pollination syndrome of *Chondroscaphe* could be similar to that of *Warscewiczella* (treated as *Cochleanthes* Raf.). However, the only species in the genus whose pollination has been documented so far, *Chondroscaphe embreei* (Dodson & Neudecker) C. Rungius, lacks the tubular or reflexed sepals that deceive pollinators as false nectaries. Instead, these are spreading, as are the fimbriate petals and lip (Fig. 8B). The lip is bilobed, with the base parallel to the column to form a gullet, then spreading and flaring in the apical half. In *C. embreei*, as well as in most species of the genus, the lip is deeply and irregularly fimbriate, but in *Chondroscaphe* species recorded in Costa Rica, it is only wavy to ruffled, with entire margins (Pupulin 2005). *Chondroscaphe embreei* is pollinated by male euglossine bees, *Euglossa trinotata*, which receive the pollinarium on the right metasoma (Reynolds 2004). The pollinarium of *C. embreei* is large, over 7 mm long, with two pairs of unequal pollinia—the adaxial ones linear and the abaxial ones linear–semi-falcate and much longer. They are connected by yellow caudicles to a broadly rhombic, erect stipe, in turn affixed to a comparatively small, obpeltate, hyaline viscidium (Fig. 9A). Even though this is the basic structure of *Chondroscaphe* pollinaria, specific variations occur. In Costa Rica, the pollinaria of *C. atrilinguis* Dressler (Fig. 9B) and *C. yamilethiae* Pupulin (Fig. 9C) are similar to that of *C. embreei*, but *C. bicolor* (Rolfe) Dressler has a much smaller, subquadrate stipe and a comparatively small, elliptic viscidium (Fig. 9D).

We cannot know which of the two parent species donated the pollen and which was fertilized. However, since the flower visitor of *Benzingia reichenbachiana* is a small bee, it would hardly be able to deposit the pollen of this species on the stigma of the *Chondroscaphe* flower, which is located in a much higher position relative to the callus of the labellum. Available information indicates that the flowers of *Chondroscaphe endresii* have an intense, spicy fragrance, suggesting the presence of compounds such as eugenol, which is the main reason male euglossine bees visit them in search of scents for their courtship ceremonies. The gullet-shaped flower of *B. reichenbachiana*, with its nectariform lateral sepals, likely also exhibits a deceptive pollination syndrome. It is possible that the inadvertent hybridization of the two species is the result of the simultaneous presence of two different pollination syndromes, in which one insect may have visited one flower for its scent and subsequently the other in search of a food reward.

## Supplementary Material

XML Treatment for Benzoscaphe

XML Treatment for Benzoscaphe stelleri

## ﻿Additional information

### Use of AI

No use of AI was reported.

### Author ORCIDs

Franco Pupulin https://orcid.org/0000-0001-6780-8395

Adam P. Karremans https://orcid.org/0000-0001-5987-7710

Elvira Salas Hidalgo https://orcid.org/0000-0002-6342-421X

### Data availability

All of the data that support the findings of this study are available in the main text.
